# Multiscale Modeling of Structure, Transport and Reactivity in Alkaline Fuel Cell Membranes: Combined Coarse-Grained, Atomistic and Reactive Molecular Dynamics Simulations

**DOI:** 10.3390/polym10111289

**Published:** 2018-11-20

**Authors:** Dengpan Dong, Weiwei Zhang, Adam Barnett, Jibao Lu, Adri C. T. van Duin, Valeria Molinero, Dmitry Bedrov

**Affiliations:** 1 Department of Materials Science and Engineering, University of Utah, 122 South Central Campus Drive, Room 304, Salt Lake City, UT 84112, USA; cherrydons@hotmail.com; 2 Department of Mechanical and Nuclear Engineering, Pennsylvania State University, University Park, PA 16802, USA; wzz2@psu.edu (W.Z.); acv13@engr.psu.edu (A.C.T.v.D.); 3 Department of Chemistry, The University of Utah, 315 South 1400 East, Salt Lake City, UT 84112, USA; adam.barnett@utah.edu (A.B.); jibao.lu@siat.ac.cn (J.L.); valeria.molinero@utah.edu (V.M.)

**Keywords:** reactive molecular simulations, atomistic and coarse-grained models, multiscale molecular simulations, alkaline fuel cells, polymer membranes

## Abstract

In this study, molecular dynamics (MD) simulations of hydrated anion-exchange membranes (AEMs), comprised of poly(*p*-phenylene oxide) (PPO) polymers functionalized with quaternary ammonium cationic groups, were conducted using multiscale coupling between three different models: a high-resolution coarse-grained (CG) model; Atomistic Polarizable Potential for Liquids, Electrolytes and Polymers (APPLE&P); and ReaxFF. The advantages and disadvantages of each model are summarized and compared. The proposed multiscale coupling utilizes the strength of each model and allows sampling of a broad spectrum of properties, which is not possible to sample using any of the single modeling techniques. Within the proposed combined approach, the equilibrium morphology of hydrated AEM was prepared using the CG model. Then, the morphology was mapped to the APPLE&P model from equilibrated CG configuration of the AEM. Simulations using atomistic non-reactive force field allowed sampling of local hydration structure of ionic groups, vehicular transport mechanism of anion and water, and structure equilibration of water channels in the membrane. Subsequently, atomistic AEM configuration was mapped to ReaxFF reactive model to investigate the Grotthuss mechanism in the hydroxide transport, as well as the AEM chemical stability and degradation mechanisms. The proposed multiscale and multiphysics modeling approach provides valuable input for the materials-by-design of novel polymeric structures for AEMs.

## 1. Introduction

The research interest in fuel cells (FCs), containing either proton-exchange membranes (PEMs) or anion-exchange membranes (AEMs), has been revived in the past decade, specifically, driven by the increasing demand for clean and renewable energy [1,2,3,4,5,6]. The advantages of alkaline fuel cells (AFCs) over proton-conducting fuel cells (PFCs) are the use of non-platinum metals (cobalt, nickel, silver etc.) as catalysts on the electrodes [6,7,8]. Typically, AEMs for AFCs are comprised of poly(p-phenylene oxide) (PPO) or polysulfone as backbone polymers, functionalized either with quaternary ammonium, imidazolium, spiroammonium, phosphonium, pyrrolidinium, guanidinium, or piperidinium [9]. In AEMs, OH^−^ is the primary charge carrier. Despite the long time since the original AFCs being developed and successfully used for Apollo projects in 1960s, numerous challenges exist advancing the AFC technology, including the search for AEM structures that have an improved chemical stability at high temperatures, efficient OH^−^ transport mechanism and desired mechanical and thermal stability [2,10,11]. Theoretical and experimental efforts have been focusing on understanding of thermodynamics, mechanical properties and chemical stability. By varying the length of alkyl tails in quaternary ammonium side groups, researchers found that hydrophobic chains can lead to the formation of larger water-rich domains/channels inside the membranes, while modification of cationic side groups with hydrophilic chains can assist the formation of continuous water channels [4,11,12]. Alternative routes to systematically improve properties of AEMs include building cross-linked structures, where the crosslinks can be either charge-neutral or built with inherent cationic nitrogen groups. Both approaches yielded significantly enhanced conductivity and mechanical robustness [13,14,15,16,17]. The AEMs mentioned above are typically made of block-copolymers. Nevertheless, non-blocky counterparts also show an enhanced ionic conductivity and electrochemical performance by allowing a higher ion-exchange capacity [18,19]. The non-blocky PPO-based AEM functionalized with quaternary ammonium will be the test polymer structure considered in this study.

Despite the ceaselessly ongoing development of novel structures for AEMs, the availability of in-depth theoretical analysis of structure-property relationships is extremely limited. The main reasons which account for such difficulties include the diversity of possible architectural designs of AEMs, multiscale heterogeneity in the morphology of hydrated membrane and the coupling of multiple physical/chemical phenomena on different length and time scales. The pioneering molecular dynamics (MD) simulation study of a hydrated AEM was done by Merinov et al. [20]. However, due to the limited spatial and time scale accessible to those simulations, the accurate diffusion mechanism and equilibrium water uptake could not be determined. By utilizing multistate empirical valence bond model, Chen et al. investigated transport mechanisms in the hydrophilic domain (poly(vinyl benzyl trimethylammonium)) of a typical block-copolymer membrane [21]. The vehicular mechanism was estimated to contribute about 80% to the total OH^−^ diffusion [21,22]. However, our recent joint reactive and non-reactive MD simulation study of non-blocky AEMs with alkylammonium functional groups demonstrated the importance of the Grotthuss mechanism in the overall transport of OH^−^, especially in assisting the OH^−^ to diffuse through narrow regions in water channels, that is the bottlenecks [23]. The chemical stability and degradation mechanisms of PPO-based AEMs have been studied recently by Zhang et al. using ReaxFF model [24,25]. Simulations using non-reactive Atomistic Polarizable Potential for Liquids, Electrolytes and Polymers (APPLE&P) force field provided accurate prediction of hydrophobicity/hydrophilicity of quaternary alkylammonium as well as dynamical properties in related aqueous solutions and AEMs [12,26]. The MD simulations based on coarse grained (CG) models of related systems have demonstrated the capability to explore thermodynamic properties of hydrated membranes, including prediction of equilibrium water uptake and quick access to equilibrated morphology of hydrated membranes [22,27]. 

While simulations using each model mentioned above provided valuable insight into subset of AEM properties, none of them is capable to address the full spectrum of characteristics needed for guiding the design and performance assessment of novel AEMs. The capabilities of three different models are summarized in Table 1. Clearly that in order to address the plethora of possible chemical events (OH^−^ transport, polymer degradation, formation of carbonate anions from CO_2_ contamination, reaction with/between other fuel/product contaminants, etc.) one has to rely on atomistically detailed methods such as mentioned above ReaxFF or on MD/quantum mechanical (QM) coupling approach as has been successfully demonstrated in recent works [28,29]. However, these methods are computationally expensive and therefore are limited to relatively small size systems and observation time scales. Hence, they can hardly access the length and time scales needed for sampling polymer chain self-assembly and microphase segregation during AEM morphology formation. On the other hand, CG models can sample the necessary length and time scales to predict AEM morphology by compromising on the accuracy of local (atomic scale) structure and dynamics and ignoring possible reaction mechanisms. An approach that is able to combine atomistic reactive, atomistic non-reactive and coarse-grained models can provide a solution to the urgent need for understanding the interplay between AEM morphology and transport processes, degradation mechanisms and chemical stability. In this work, we demonstrate how three different models can be combined in a joint multiscale and multi-physics approach. 

## 2. System Description and Methodology

### 2.1. System

**Figure 1:**
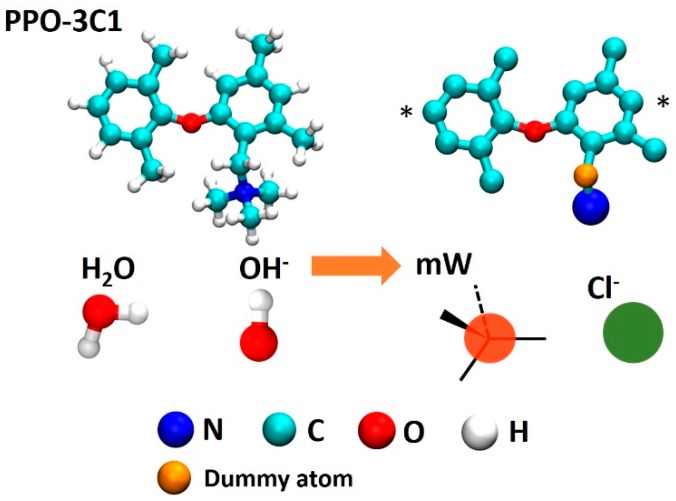
Structure of molecules/ions simulated in this study: with atomistic (**left**) and coarse-grained (**right**) representations.

The molecular structure of simulated molecules, including PPO-based segment, hydroxide and water molecules are shown in Figure 1. To demonstrate the multiscale approach, we have selected a relatively small size system for which we can access long time scales using all three techniques. Specifically, our system contains 16 polymer chains each comprised of 10 monomers and every other monomer functionalized with tetramethyl ammonium(TMA) cationic group, as shown in Figure 1, that is, 5 functional groups per chain. The polymer chains were mixed with 800 water molecules and 80 anions, therefore resulting in the degree of hydration λ= 10 (ten water molecules per each cationic group), which is a typical hydration level for PPO-based membranes. In atomistic simulations, the anion was OH^−^ while in the CG simulations, it was Cl^−^. Since the anion is mostly dissociated from cationic groups in such membranes, we do not expect that substitution of OH^−^ for Cl^−^ in the CG model would have a significant influence on the overall membrane morphology. 

### 2.2. Multiscale Modeling Approach

**Figure 2:**
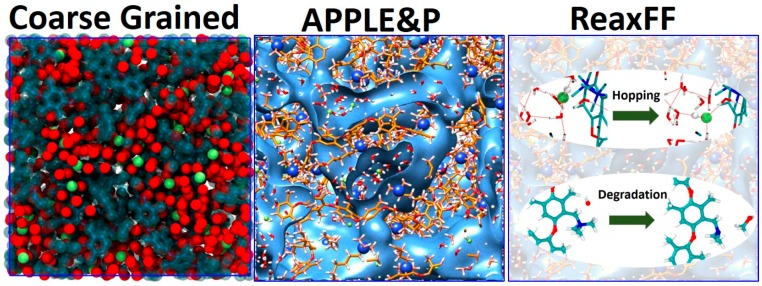
Illustration of combining simulations at different scales. The red dotted lines in the right panel indicate hydrogen bonds.

Within the proposed combined approach, the equilibrium morphology of hydrated AEM is obtained using the CG simulations [30]. As we showed in previous studies, the membranes of PPO-based polymers functionalized with quaternary ammonium undergo microphase segregation, forming water-rich domains/channels and polymer-rich glassy domains [11,12]. Hence, once the membrane morphology is formed using CG simulations, its three-dimensional structure will likely be preserved after its mapping and simulations using atomistic models. Therefore, the morphology of AEM from the CG model was then mapped to the APPLE&P model to study the local hydration structure of ionic groups, vehicular transport mechanisms and distribution of water molecules inside channels in the membrane. Subsequently, this equilibrated atomistic configuration was mapped to the ReaxFF model, with which we sampled contributions of the Grotthuss mechanism to the hydroxide transport, as well as the chemical stability and degradation mechanisms of the membrane. The focus of three different models is illustrated in Figure 2. Below we provide the description of simulation details, while in the next section we discuss and compare properties predicted by simulations at each level of resolution.

### 2.3. Coarse Grained Simulations

The coarse-grained model of the hydrated PPO/TMA membrane was presented in reference [22]. The parameterization of the coarse-grained model used in this study have been derived and verified in a previous study [22]. The coarse-grained mW model was used to represent water [28]. The tetramethylammonium cationic group has been coarse-grained into one chargeless particle, while anion (Cl^−^) was represented with single non-charged force center [31]. In this work we used Cl^−^ as anion for the following reasons: 1) several properties of investigated PPO-based AEM, such as, for example, the water uptake, have been characterized experimentally with Cl^−^ anion and therefore substantial development of the CG model was done based on comparison with those experiments; and 2) due to high degree of dissociation of anions from cationic group, we believe that for the formation of membrane morphology (the main property extracted from the CG model) is not influenced by the nature of anion (as long as we are dealing with small anions). The interactions between TMA-Cl and TMA-mW were described by the two-body part of Stillinger-Weber potential as well as a short-ranged Yukawa potential to mimic the shielded coulomb interactions [32]. The standard 12-6 Lennard-Jones potential with the taper function was utilized to calculated repulsion-dispersion interaction, in which the inner and outer cut-off radius was set to be 7.0 Å and 10.0 Å, respectively. The models for backbone of PPO were fully represented using united atoms with hydrogen atoms collapsed on corresponding carbons.

The CG simulations were conducted with LAMMPS [33], integrating the equations of motions with the velocity Verlet algorithm with a time step between 0.5 fs at 2000 K to 3 fs at 298 K. The temperature and pressure were controlled with a Nose-Hoover thermostat and barostat with relaxation times equal to 100 and 4000 of the corresponding time step, respectively.

To create the equilibrated nanosegregated structure of the hydrated polymer membrane, we follow the procedure of ref [22]. First, an initial low-density (0.01 g cm^−3^) amorphous structure of the PPO/TMA oligomers and water was created using Scienomics’ Materials and Process Simulation Platform (MAPS). Then, the structure was then compressed under 1 atm, resulting in a randomly distributed simulation box of polymer and water. This box was then equilibrated in the *NPT* ensemble at 1 atm and 500 K for 10 ns before being cooled down linearly to 298 K over an additional 10 ns to ensure that the simulation box has a stable volume at room temperature and pressure. Once a stable volume was achieved, the simulation box was then annealed at 2000 K in *NVT* simulations at that temperature over 5 ns to create a further equilibrated structure that accurately represented the nanophase segregation of the PPO-TMA membrane. The resulting structure then underwent a two-step process of cooling and equilibration to bring the simulation back to room temperature and pressure. The first of these steps sets the temperature to 500 K and ramped it back down to 298 K over 10 ns, then evolved the cooled system for an additional 5 ns. The finalized membrane was then evolved in *NpT* simulations 298 K and 1 atm to determine membrane properties.

### 2.4. Atomistic Non-Reactive Simulations (APPLE&P)

The APPLE&P force fields has been parameterized and developed following procedure as described in reference [34]. The ability of this force to describe alkyl-ammonium cations in aqueous solutions, especially the dynamical and thermodynamic properties, has been verified in our previous study [26]. The parameters for the description of the PPO backbone and hydroxide have been derived and discussed previously as well [12]. In simulations with the APPLE&P force field, all chemical bonds were constrained using a modified SHAKE algorithm [35]. For the van der Waals and real-space electrostatic interactions, a 15.0 Å cut-off radius was used. Ewald summation method [36] was used for the calculation of electrostatic interaction in the reciprocal space. At each atomic center, the isotropic point polarizability was assigned to mimic the response of charge cloud to the local electric field induced by neighboring point charges. The interactions between charges and induced dipoles were also calculated with Ewald summation method, while interactions between induced dipoles were smoothly truncated within 15.0 Å cut-off radius. A multiple time step integration technique has been used, with the unit integration step of 0.5 fs used for intramolecular bonded degrees of freedom, a 1.5 fs time step for short-range non-bonded interaction within 8.0 Å cut-off radius and a 3.0 fs time step for the remaining nonbonded interaction and Ewald summation in reciprocal space.

The AEM configuration equilibrated using CG model has been mapped to the APPLE&P model as following: First, the hydrogen atoms in the polymer backbone which are not represent in the CG model were added in consistence with the expected C–H bond length and bend angles defined in the APPLE&P force field. The fully atomistic representation of TMA-like functional groups was added by putting the center of TMA to the location of the CG representation of the cationic group. Atomistic OH^−^ was added by putting the O in OH^−^ in the location of Cl^−^ in the CG model while orientations of the O–H bond were randomly chosen. After that, the system was equilibrated for 2 ns to allow relaxation of local structure and conformations. Production simulations using APPLE&P model were conducted over 30 ns in the *NPT* ensemble using the Nose-Hoover thermostat and barostat.

### 2.5. Atomistic Reactive Simulations (ReaxFF)

The ReaxFF simulations were run with the reparameterized “CHON-2017_weak” force field, which showed good description of weak interactions of functionalized hydrocarbon/water [37]. Because both APPLE&P and ReaxFF models have fully atomistic representation the mapping configurations from APPLE&P to ReaxFF is straightforward and only minor relaxation in the local structure was required. After mapping of configuration, ReaxFF simulations with *NVT* ensemble were conducted for 600 ps using ADF simulation package [38]. Thereafter, a production trajectory over 500 ps long was obtained for further analysis. In the study of the chemical degradation, the simulation temperature was increased to 500 K, to increase the rate of events of chemical reactions in the simulation cell. The simulations were conducted with a 0.25 fs time step. The temperature was controlled with Berendsen thermostat with damping constant of 100 fs.

## 3. Results and Discussion

The multi-scale modeling starts with equilibration of membrane morphology using the CG model. We rely on the accuracy of CG model to capture thermodynamically driven microphase segregation as well as its ability to access long simulation times to allow relaxation of polymer degrees of freedom and morphology ripening. Subsequently, this morphology is mapped to the atomistic non-reactive model (APPLE&P) and additional simulations are conducted at this level to equilibrate the local hydration structure of ions and distribution of water inside water channels. After that, configurations obtained from APPLE&P simulations are mapped to the ReaxFF model to sample reactions and Grotthuss transport of hydroxide on short time and length scales.

### 3.1. Morphology of the Hydrated Membrane

Taking into account that PPO is a glassy polymer at room temperature, we anticipate that the underlying membrane morphology is preserved after mapping from one model to another. This assumption is tested by examination of the distribution of cross-sectional widths of the water channels formed in the membrane, estimated by analyzing the distribution of sphere diameters which can fit inside random location inside the water channel boundary (defined as 25% of bulk water density), which is consistent with the process in our previous studies [12,23].

**Figure 3:**
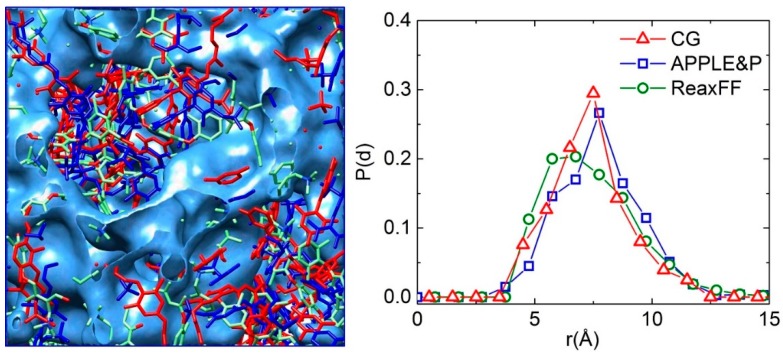
Left panel: Snapshot of morphology of hydrated membrane (red for polymer phase using coarse grained (CG) model; blue for polymer phase using APPLE&P, green for polymer phase from ReaxFF model; blue isosurface of water channels are drawn at 50% of bulk water density). Right panel: Distribution of water channel sizes from different models.

The AEM morphology at hydration level *λ* = 10 is illustrated on the left panel of Figure 3 after it was mapped from the CG model to APPLE&P and further to ReaxFF. While we observe some local displacement of polymer segments, the overall morphology of hydrated AEM after mapping to different models is conserved. Furthermore, the distribution of water channel sizes was calculated based on the concept of ‘pore size distribution’ (PSD) typically used to evaluate distributions in porous materials [39]. As shown in the right panel of Figure 3, the averaged channel width from PSDs is around 7.2 Å. Although, the PSDs are not exactly identical for different models, the overall features of distributions are consistent, including the average channel size and the overall broadness of the distribution, further validating the direct mapping of the morphology across the different models.

### 3.2. Structural Properties

**Figure 4:**
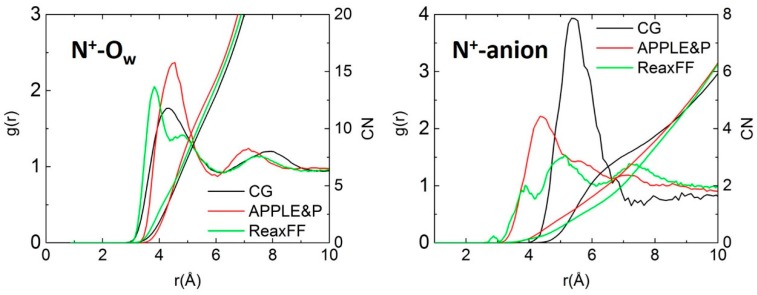
Comparison of radial distribution functions (RDF) and coordination numbers (CN) obtained from different models: (**left**) N^+^-O_w_ and (**right**) N^+^-anion.

Pair-wise structural correlations characterize local coordinating environments. The radial distribution functions (RDF, *g(r*)) of water oxygen (O_w_) and anion around cationic nitrogen (N^+^) are given in Figure 4 A and B, respectively. The apparent coordination numbers (CN), obtained by integration of the RDFs from 0 to *r*, are also shown for each model. The three models show some difference in the water hydration of the cationic group, however all of them have the first minimum in the N^+^-O_w_ RDF at ~6.1 Å, resulting in similar first hydration shells that contain about 13-15 water molecules. For the cation-anion RDF, we see more substantial differences, particularly between the CG model and atomistic simulations. This is not surprising because in the CG model not only the anion is different, Cl^−^ instead of OH^−^ but both the anion and cation are represented as single spheres which limits the possibility of closer approaches that can occur in atomistic models.

### 3.3. Dynamical Properties

Next, we compare dynamical properties predicted by three models. Specifically, we focused on the analysis of self-diffusion coefficients and the residence times of different neighboring species.

To investigate the influence of the binding between cationic nitrogen and moving species (water molecules and anions), the residence autocorrelation function (ACF) was calculated as:

(1) $$\;ACF\left(t\right)=\frac{\langle H_{\mathrm{ij}}\left(t\right)\times H_{\mathrm{ij}}\left(0\right)\rangle }{\langle H_{\mathrm{ij}}\left(0\right)\times H_{\mathrm{ij}}\left(0\right)\rangle }\;$$

where $H_{\mathrm{ij}}\left(t\right)=1$ when the moving specie *j* is within defined coordination range of cation *i*, while $H_{\mathrm{ij}}\left(t\right)=0$ when the specie diffuses out of that range. In this study, we defined the first coordination (hydration) shell of water around N^+^ based on the first peak in the N^+^-O_w_ RDF, that is, *r* < 6.1 Å. The ACF were subsequently fitted with the Kohlrausch-Williams-Watts (KWW) stretched exponential function:

(2) $$\;P\left(t\right)=A\times \exp\left(-{\left(\frac{t}{\tau_{\mathrm{KWW}}}\right)}^{\beta }\right)\;$$

The characteristic residence times were obtained by integration of Equation (3):

(3) $$\;\tau =\overset{\infty }{\underset{t=0}{\int }}P\left(t\right)dt\;$$

**Figure 5:**
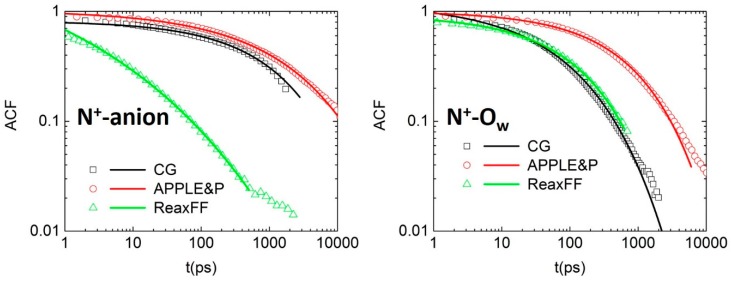
Autocorrelation functions (ACFs) and Kohlrausch-Williams-Watts (KWW) fits obtained from simulations using three models.

The ACFs obtained from simulations using three different models are shown in Figure 5. Examination of N^+^-anion correlation indicates that non-reactive models (CG and APPLE&P) predict two orders of magnitude longer residence time of the anion near cationic group compared to ReaxFF. The residence of OH^−^ around N^+^ can influence the chemical stability and degradation rates of AEMs during operation. Previous experimental study demonstrated that longer alkyl chains attached to cationic group can reduce the residence time of OH^−^ near N^+^, therefore protecting it from OH^−^ attack and chemical degradation [3,4]. Therefore, this characteristic and its apparent difference between models is quite important. From Figure 4 it is clear that in all models the anion has a preference to be within the first hydration shell of cationic groups, which is consistent with relatively low hydration level of the membrane. In the CG and APPLE&P models, in order to leave the hydration shell of the cation, the anion has to rely on vehicular motion. Moreover, due to strong coordination of anion with water, it would likely involve the anion moving together with some of the hydrating water molecules. The diffusion of such anion-water clusters requires a relatively large activation energy. On the other hand, when introducing Grotthuss hopping in ReaxFF, the OH^−^ diffusion from inside to outside of the range of the first hydration shell of cationic group can be easily achieved through a couple of steps of proton hopping between OH^−^ and water molecules, without the need to drag the anion hydration along. This leads to a much faster decay of the anion residence times near cationic groups and likely represents the most realistic description of this process in AEM.

For the residence time of water near N^+^ we see a less pronounced difference between the three models, with the APPLE&P showing the slowest relaxation and the CG and ReaxFF showing a faster decay, albeit for different reasons. In ReaxFF, the presence of Grotthuss hopping of OH^−^ also speeds up the water dynamics in the anion surrounding [23]. In the CG model, the single site representation of TMA cationic group, does not allow for water to experience the chemical “corrugation” of this group and, in consequence, the overall dynamics speeds up, a phenomenon typically observed for all coarse-grained models. The resulting characteristic residence times are summarized in Table 2. In simulation with the non-reactive models, the decay of N^+^-O_w_ correlation is faster than N^+^-anion correlation, giving the ratio $\frac{\tau_{N^{+}-\mathrm{anion}}}{\tau_{N^{+}-O_{w}}}\;$of these residence times of 9.5 and 4.0 in the CG and APPLE&P simulations, respectively. However, a reversed trend is observed in ReaxFF, in which this ratio is 0.38. The introduction of Grotthuss hopping shows a strong impact on the residence time ratio between anion and water molecules.

The diffusion coefficient of molecules was calculated based on the Einstein relation:

(4) $$\;MSD=\langle \frac{1}{M}\overset{M}{\underset{m=1}{\sum }}{\left(r_{m}\left(t\right)-r_{m}\left(0\right)\right)}^{2}\rangle =6Dt\;$$

in which the *MSD* is the mean square displacement, *M* is the total number of molecules of the species of interest, $r_{m}\left(t\right)$ is the location of molecule *m* at time *t*, *D* is the self-diffusion coefficient and < > brackets indicate the averaging over multiple time origins available from the trajectory for a given time interval *t*. In this study, only ReaxFF simulations included the Grotthuss mechanism, therefore resulting contributions to diffusion from both vehicular motion and Grotthuss hopping. In simulations with APPLE&P and CG force fields, the diffusivity of anion and H_2_O was solely the result of vehicular mechanism, although the CG model tried to capture the total diffusivity, while APPLE&P has focused on capturing contributions from vehicular motion [12,22]. Due to a non-reactive OH^−^ representation in the APPLE&P, the vehicular motion of OH^−^ in bulk water accounts for ~40% of experimental diffusion [26], in consistence with the value obtained from charge-ring model and reactive Lewis model [40,41]. In the CG model, the anion force field was developed to preserve the ratio of the chloride to water diffusion in reference non-polarizable atomistic simulations of TMACl solutions [27]. Following the Einstein relation in Equation (4), the self-diffusion coefficient of H_2_O and anion in the membrane and in dilute aqueous solution (i.e., bulk water-like environment) are shown in Table 3 for three different models.

In bulk water, all models capture the diffusion of water molecules predicted by all models is in good agreement with experimental data. For the anion diffusion, we see significant influence of the model. In the CG model, the anion (Cl^−^) diffusion is about factor of 2.2 lower than *D*_H2O_. Simulations with the APPLE&P, predict OH^−^ slightly smaller (~20%) than *D*_H2O_. Both in the CG and APPLE&P models, there is only vehicular mechanism for anion transport and hence both of these models significantly underestimate experimental value for OH^−^ diffusion. For example, in the APPLE&P, the diffusion of OH^−^ in bulk water is 0.19 Å^2^ ps^−1^ at room temperature, which is ~40% of the experimental value at 0.53 Å^2^ ps^−1^ [42]. Note, that in experiment, due to presence of the Grotthuss hopping, the self-diffusion of OH^−^ is larger than diffusion of water by factors of 2.2, while predictions of CG and APPLE&P non-reactive models predict the ratio *D*_anion_*/D*_H2O_ < 1.0. In ReaxFF, which does include the Grotthuss mechanism explicitly, the diffusion of OH^−^ is significantly higher than in non-reactive simulations and the ratio *D*_anion_*/D*_H2O_ = 5.9, qualitatively consistent with experimental observations.

In the hydrated membrane, the inter-connected sub-nanometer water channels define the overall morphology of the membrane. Due to the hydrophobic interaction between polymer and water [26], together with the enhancement of hydrophobicity by confined environment [43], a significant decrease in the dynamics of H_2_O and anion is observed in all models. As can be seen from Table 3, the diffusion of H_2_O and anion predicted by all models significantly decrease in the membrane, yet the ratio *D*_anion_*/D*_H2O_ remain qualitatively the same as in bulk water. In ReaxFF the ratio is 5.7 and is very similar to bulk water, while in the CG and APPLE&P models it reduces even further and becomes 0.18 and 0.30, respectively. We note that the introduction of the hopping mechanism in ReaxFF creates additional perturbations (during hopping events) to the local water hydrogen-bonding network inside water nanochannels. While the CG and APPLE&P simulations predict similar *D*_anion_*/D*_H2O_ ratios, the absolute values of diffusion coefficients are noticeably different. The CG values are factor of 6 to 9 higher than in the APPLE&P. But anion mobility in both is still much lower than the values from ReaxFF that includes both the vehicular motion and Grotthuss mechanism.

To understand the OH^−^ transport mechanism, the MSD of OH^−^ from ReaxFF simulation can be decomposed into contributions from the vehicular and Grotthuss motions. The general decomposition of MSD can be defined in the following form:

(5) $$\;r_{n,0\to t}=\underset{t_{1}}{\sum }P\times \left(r_{n,t_{1}\to t_{1}+\Delta t}\right)$$

where Δt is an integration step, the binary function *P* is defined in Equation (6):

(6) $$\;P=(\begin{array}{c} \;\;0,\;\;\;\;\;\;\;\;\mathrm{Grotthuss}\;\;\\ 1,\;\;\;\;\;\;\;\;\mathrm{vehicular} \end{array}\;$$

**Figure 6:**
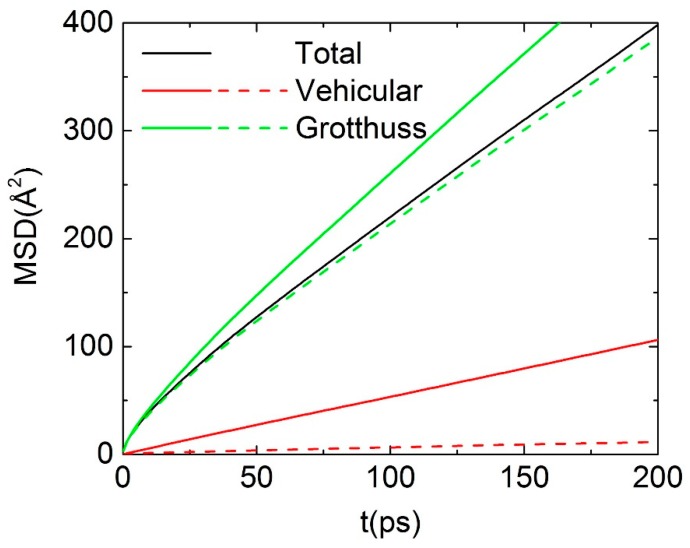
Decomposition of MSD of OH^−^ into Grotthuss hopping and vehicular contribution (ReaxFF only). The dashed lines indicated the net contribution from corresponding diffusion mechanism.

Therefore, the total MSD ($MSD_{\mathrm{tot}})$ can be decomposed into:

(7) $$\;MSD_{\mathrm{tot}}=MSD_{c}+MSD_{d}+2\times MSD_{\mathrm{cd}}\;$$

where $MSD_{c}$ denotes the MSD of continuous vehicular motion, $MSD_{d}$ denotes MSD of discrete Grotthuss hopping and $MSD_{\mathrm{cd}}$ is a contribution from correlational Grotthuss-vehicular coupling. Consistent with the previous studies [21,23], the net contribution from vehicular motion and Grotthuss hopping to the total anion diffusivity can be defined as $MSD_{c}+MSD_{\mathrm{cd}}$ and $MSD_{d}+MSD_{\mathrm{cd}}$, respectively. Different from the method to identify the location of hydroxide based on the center of excess charge (CEC), the method we used here is based on assigning hydrogen (H) to the nearest oxygen (O) atom. Afterwards, the number of H bound to each O is calculated. The assignment of whether the O belongs either to OH^−^ or H_2_O can be clearly identified, that is, O bonded with one H is identified as OH^−^, while bonding to two H atoms will lead to identification as H_2_O [23]. Following this identification method, the decomposed MSD has been obtained from ReaxFF simulations and are shown in Figure 6. For the hydrated membrane investigated here, the Grotthuss hopping is the dominant mechanism to the total diffusion of OH^−^. If considering the effects of vehicular-Grotthuss coupling, the net contribution from vehicular motion is less than 5% [23].

### 3.4. Degradation Mechanism of AEM (ReaxFF)

**Figure 7:**
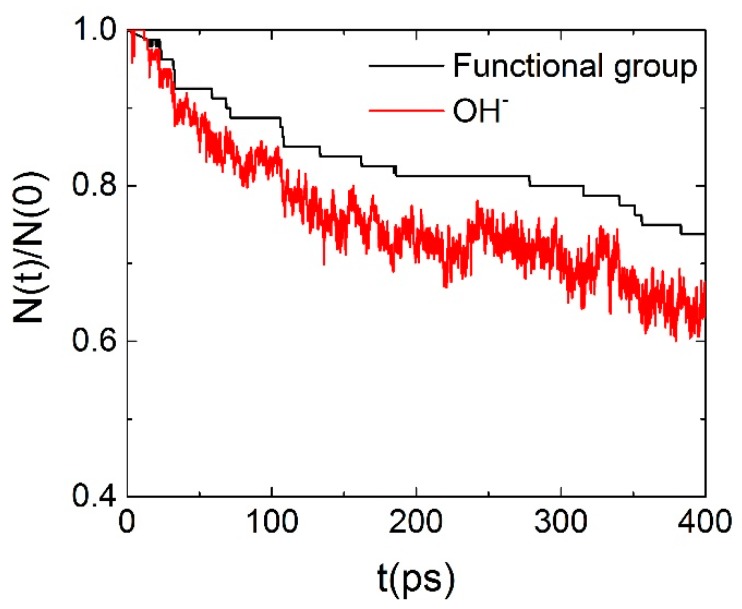
Time dependence of residual ratios for functional cationic groups and OH^−^.

Simulations using ReaxFF also allowed us to study chemical stability of the AEM, by monitoring the consumption of OH^−^ and degradation of polymer backbone/functional groups. At 300 K, no degradation of polymer was observed over the time scale accessible to simulations (~ns). In order to speed up the reaction processes and to observe degradation, ReaxFF simulations were conducted at 500 K. The time dependence of the fractions of unreacted cationic groups and OH^−^ remaining in the system are illustrated in Figure 7.

**Figure 8:**
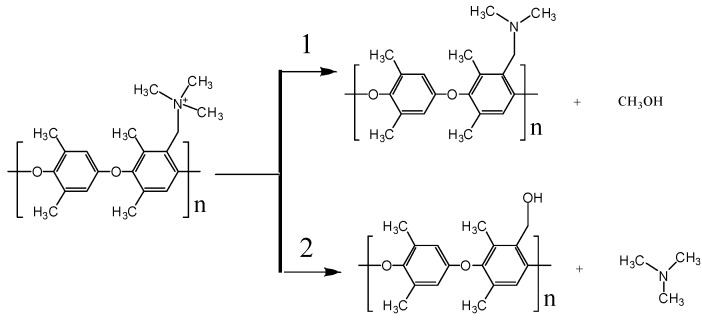
Degradation mechanism in PPO-3C_1_ hydrated membrane observed in ReaxFF simulations.

Based on the observed reaction steps leading to the OH^−^ consumption and reactivity of functional groups, the reaction mechanisms illustrated in Figure 8 were observed in ReaxFF simulations. In our simulations, we see two primary pathways: An OH^−^ can attack methyl groups bonded to cationic nitrogen and forms CH_3_OH and charged neutral amine, which follows an ylide reaction mechanism [37]. Alternatively, an OH^−^ can react with methylene bridge, forming alcohol with polymer and free amine. We did not observe a degradation of the polymer backbone, however that could be just a kinetic issue and would require much longer simulation times.

## 4. Conclusions

Molecular dynamics simulations of hydrated PPO-based membranes have been conducted using three models with different resolution in interaction and particle spaces: nonreactive coarse-grained (CG) model, atomistic nonreactive (APPLE&P) and reactive (ReaxFF). Despite the difference in models representations, CG/APPLE&P/ReaxFF can be coupled together to investigate systems with complex structure-property correlations and to sample complementary properties. The CG and APPLE&P models have capabilities to efficiently obtain the morphology of hydrated membrane, accurately capturing thermodynamics and vehicular motion of different species, while ReaxFF can use those morphologies as an input and sample the Grotthuss motion and reactivity in AEMs. In the investigated system, with a network of narrow (subnanometer wide) water channels, the charge transport is a strong function of hydrophobicity of cationic group and details of water channel geometry. We demonstrate that in such environments the Grotthuss hopping mechanism plays an important role in the charge transport and hence should be included into consideration. The proposed multiscale modeling approach illustrates how different force fields/models can be coupled to provide complementary properties and access the full spectrum of characteristics needed for material-by-design approach of AEMs and other related systems with multiscale and multiphysics phenomena.

## Figures and Tables

**Table 1:** The capability of different simulation models in retrieving properties of hydrated anion exchange membrane (AEM) *.

Capability	Reactive Atomistic MD	Non-Reactive Atomistic MD	Coarse Grained MD
Water uptake	-	?	✔
Nanoscale morphology of short oligomers	✔	✔	✔
Nanoscale morphology of long chains	-	-	✔
Local dynamics (a few ns timescale)	✔	✔	?
Global dynamics (up to microsecond)	?	✔	✔
Thermodynamics	✔	✔	✔
Local structure	✔	✔	-
Grotthuss mechanism	✔	-	-
Membrane chemical stability	✔	-	-

* ‘✔’ definitely capable, ‘-‘ incapable, ‘?’ possibly capable.

**Table 2:** Characteristic residence times (in ps) of anion and H_2_O and their ratio.

	Residence Time		
	$\;\tau_{N^{+}-\mathrm{anion}}\;$	$\;\tau_{N^{+}-O_{w}}\;$	$\tau_{N^{+}-\mathrm{anion}}$ $/\tau_{N^{+}-O_{w}}\;$
CG	934.5 *	98.0	9.5
APPLE&P	2285.6	567.5	4.0
ReaxFF	38.9	102.6	0.38

* Anion is Cl-.

**Table 3:** Diffusion coefficient of anion and H_2_O and the corresponding ratio.

	Diffusion Coefficient in Membrane			Diffusion Coefficients in Bulk Water		
	*D*_OH_^−^ (Å^2^/ps)	*D*_H2O_ (Å^2^/ps)	*D*_OH_^−^/*D*_H2O_	*D*_OH_^−^ (Å^2^/ps)	*D*_H2O_ (Å^2^/ps)	*D*_OH_^−^/*D*_H2O_
EXP	-	-	-	0.53	0.23	2.3
CG	0.0140 *	0.0770	0.18	0.30 *	0.65	0.46
APPLE&P	0.0025	0.0083	0.30	0.19	0.23	0.81
ReaxFF	0.2980	0.0521	5.72	1.48	0.25	5.9

* Anion is Cl^−.^
